# Investigating the association of atopic dermatitis with ischemic stroke and coronary heart disease: A mendelian randomization study

**DOI:** 10.3389/fgene.2022.956850

**Published:** 2022-08-30

**Authors:** Jian Huang, Ying Gui, Jing Wu, Yubo Xie

**Affiliations:** ^1^ Clinical Laboratory Center, The First Affiliated Hospital of Guangxi Medical University, Nanning, China; ^2^ Department of Anesthesiology, The First Affiliated Hospital of Guangxi Medical University, Nanning, China; ^3^ Guangxi Key Laboratory of Enhanced Recovery After Surgery for Gastrointestinal Cancer, The First Affiliated Hospital of Guangxi Medical University, Nanning, China

**Keywords:** atopic dermatitis, ischemic stroke, coronary heart disease, Mendelian randomization, myocardial infarction

## Abstract

**Background:** Atopic dermatitis (AD) is the most common chronic skin inflammatory disease. Prior observational studies have reported inconsistent results on the association of AD with ischemic stroke and coronary heart disease. In this study, we applied two-sample Mendelian randomization (MR) to evaluate the causal effect of AD on ischemic stroke and coronary heart disease.

**Methods:** Twelve single-nucleotide polymorphisms robustly associated with AD (*p* < 5 × 10^–8^) were obtained from a genome-wide association study that included 10,788 cases and 30,047 controls by the EArly Genetics and Life course Epidemiology (EAGLE) Consortium (excluding the 23andMe study). The corresponding data for ischemic stroke (34,217 cases and 406,111 controls), large artery stroke (4,373 cases and 406,111 controls), cardioembolic stroke (7,193 cases and 406,111 controls), small vessel stroke (5,386 cases and 192,662 controls), coronary heart disease (122,733 cases and 424,528 controls), and myocardial infarction (43,676 cases and 128,199 controls) were obtained from the MR-Base platform. In the primary MR analyses, we applied the inverse variance weighted method to evaluate the associations. We performed a sensitivity analysis using weighted median, MR-Egger, weighted mode, simple mode, Mendelian Pleiotropy RESidual Sum and Outlier (MR-PRESSO), and leave-one-out methods.

**Results:** In the primary MR analyses, we found no causal association of genetically predicted AD with ischemic stroke [odds ratio (OR) = 1.00, 95% confidence interval (CI): 0.95–1.06], large artery stroke (OR = 1.02, 95% CI: 0.88–1.17), cardioembolic stroke (OR = 1.06, 95% CI: 0.94–1.18), small vessel stroke (OR = 1.05, 95% CI: 0.94–1.17), coronary heart disease (OR = 1.00, 95% CI: 0.94–1.05), and myocardial infarction (OR = 1.03, 95% CI: 0.98–1.09). The results from the primary MR analyses were supported in sensitivity analyses using the weighted median, weighted mode, simple mode, and MR-Egger methods and multivariable MR analyses adjusting for asthma and several traditional risk factors for ischemic stroke and coronary heart disease. MR-Egger intercepts provided no evidence of directional pleiotropy. The MR-PRESSO and leave-one-out analyses did not indicate any outlier instruments.

**Conclusion:** Our MR study does not support a causal association of genetically predicted AD with ischemic stroke, large artery stroke, cardioembolic stroke, small vessel stroke, coronary heart disease, and myocardial infarction.

## Introduction

Atopic dermatitis (AD), also known as eczema, is a chronic and relapsing inflammatory skin disorder. It affects 10%–20% of children and up to 10% of adults (Hadi et al., 2021). AD is characterized by eczematous lesions, intense pruritus, and helper cell type 2 (Th2)-dominated immune responses in the skin (Hadi et al., 2021). It has long been known that AD has a consistent association with other atopic and allergic conditions such as atopic rhinitis and asthma (Knudgaard et al., 2021; Yaneva and Darlenski, 2021). In recent years, increasing attention has been given to the association of AD with nonallergic conditions, such as ischemic stroke and coronary heart disease. Although a number of observational studies have been performed on this topic, conflicting results have been reported in the published literature. For instance, in the National Health and Nutrition Examination Survey (2005–2006) and the National Health Interview Survey (2010–2012), AD was found to be linked with coronary heart disease and stroke (Silverberg, 2015). In addition, an Asian study using the Korean National Health Insurance data also found that patients with AD were associated with a higher risk of myocardial infarction [hazard ratio (HR) = 9.43, *p* < 0.001] and stroke (HR = 10.61, *p* < 0.001) (Jung et al., 2021). However, positive associations were not found in German and Canadian cohorts and a large-scale meta-analysis (Drucker et al., 2017; Standl et al., 2017; Thyssen et al., 2018). It is well known that observational studies are susceptible to confounding (Lawlor et al., 2008); they cannot estimate the causal effects of AD on ischemic stroke and coronary heart disease.

Mendelian randomization (MR) is an epidemiological approach that can assess a causal effect of exposure on a disease outcome. In an MR analysis, genetic polymorphisms that are robustly associated with exposure are used as instrumental variables (Lawlor et al., 2008). Because alleles are randomly allocated during conception, MR can greatly reduce potential biases of observational studies which include residual confounding. Ischemic stroke and coronary heart disease are two common forms of cardiovascular disease which are associated with severe mortality and morbidity. In this study, we performed a two-sample MR to estimate whether there is a causal effect of AD on ischemic stroke and coronary heart disease using the genome-wide association studies (GWAS) summary data.

## Materials and methods

### Study design

We applied a two-sample MR design in the present study. Supplementary Figure S1 shows the key MR assumptions. Since we only used publicly available data sets with no involvement of participants, ethics approval was not required for this MR study.

### Summary-level data for atopic dermatitis

We obtained GWAS summary statistics from the MR-Base platform (http://www.mrbase.org/) (Hemani et al., 2018). It is a freely accessible database for MR developed by the Medical Research Council Integrative Epidemiology Unit at the University of Bristol, which contains over 40,000 GWAS summary data sets for a variety of human phenotypes. The exposure data were obtained from a GWAS that included 10,788 patients with AD and 30,047 controls by the EArly Genetics and Life course Epidemiology (EAGLE) Consortium (excluding the 23andMe study) (Paternoster et al., 2015). According to the EAGLE Consortium, AD was diagnosed by self-report or dermatological exam (Paternoster et al., 2015). Single-nucleotide polymorphisms (SNPs) associated with AD at genome-wide significance (*p* < 5 × 10^–8^) were used as instrumental variables. To ensure that each instrumental variable is independent (*r* ^2^ < 0.001), we clumped instrumental variables for linkage disequilibrium using the R package “TwoSampleMR” version 0.5.6 (https://github.com/MRCIEU/TwoSampleMR). We did not use palindromic SNPs with intermediate allele frequencies, since they may invert the direction of a causal effect. The F-statistic was computed to assess the strength of the extracted instrumental variables. It is conventionally accepted that an F-statistic value of >10 is indicative of a strong instrument (Burgess et al., 2011). All data applied were derived from participants of European ancestry.

### Summary statistics for ischemic stroke and coronary heart disease

Summary statistics for ischemic stroke was obtained from a GWAS meta-analysis which included 440,328 participants (34,217 cases and 406,111 controls) of European ancestry from the International Stroke Genetics Consortium (Malik et al., 2018). The majority of the patients with ischemic stroke received brain imaging confirmation. The causal role of AD on common etiological subtypes of ischemic stroke such as large artery atherosclerosis (4,373 cases and 406,111 controls), cardioembolic stroke (7,193 cases and 406,111 controls), and small-vessel stroke (5,386 cases and 192,662 controls) was also evaluated. Ischemic stroke subtypes were classified based on the Trial of Org 10172 in Acute Stroke Treatment criteria (Adams et al., 1993). The summary statistics for coronary heart disease was obtained from a large-scale meta-analysis of UK Biobank (UKBB) and CARDIoGRAMplusC4D data sets (122,733 cases and 424,528 controls) (van der Harst and Verweij, 2018). The summary-level data for myocardial infarction were extracted from a comprehensive GWAS meta-analysis of CARDIoGRAMplusC4D data sets (43,676 cases and 128,199 controls) (Nikpay et al., 2015). Supplementary Table S1 shows the detailed descriptions of the data sources. Proxy SNPs (at *r* ^2^ ≥ 0.8) were used if SNPs were missing in the outcome data sets. To ensure that the effect of each SNP on the outcome and exposure was relative to the same allele, we harmonized exposure and outcome data using the TwoSampleMR R package (version 0.5.6).

### Statistical analysis

In the primary MR analyses assessing the relation of AD with ischemic stroke and coronary heart disease, the inverse variance weighted (IVW) method was applied. This method generates a consistent assessment for a causal association when all selected SNPs are valid instruments (Burgess et al., 2017). When Cochran’s Q statistic was significant, a multiplicative random-effects model was applied for the inverse variance weighted method. We conducted sensitivity analyses using MR-Egger, Mendelian Randomization Pleiotropy RESidual Sum and Outlier (MR-PRESSO), weighted median, simple mode, and weighted mode methods to ensure that horizontal pleiotropy did not bias the main results. The MR-Egger method is a weighted linear regression that can evaluate the average pleiotropic effect as the intercept, where a significant deviation from zero indicates directional pleiotropy (Bowden et al., 2015; Burgess and Thompson, 2017). The MR-PRESSO method can detect and correct for outlying SNPs (Verbanck et al., 2018). The weighted median method assesses causal effects requiring at least half of the weight in the analysis stems from valid instruments (Bowden et al., 2016). The weighted model method uses clusters of valid instrumental variables for evaluating the causal relationship (Hartwig et al., 2017). MR sensitivity analyses can support the results from the main analyses (Liu et al., 2021; Tan et al., 2021; Tan et al., 2022). Cochran’s Q statistic was computed to evaluate heterogeneity between individual SNPs (Bowden et al., 2017). We conducted a leave-one-out MR analysis for ensuring that causal effects were not observed owing to the influence of a single instrumental SNP.

We performed a multivariable MR to adjust for asthma and common risk factors for ischemic stroke and coronary heart disease, namely, type 2 diabetes, hypertension, body mass index (BMI), smoking initiation, and alcohol intake frequency (Burgess and Thompson, 2015). Data sets for these confounding factors were extracted from the MR-Base platform (http://www.mrbase.org/) (Hemani et al., 2018). Supplementary Table S1 shows the characteristics of these data sets.

Statistical power calculations were performed by utilizing the online mRnd power calculator (http://cnsgenomics.com/shiny/mRnd/) developed by Brion et al. (2013). All the analyses were undertaken using the R programming language (R Core Team, Vienna, Austria. https://www.R-project.org/). The statistical significance was set to *p* < 0.05 unless otherwise specified. All confidence intervals (CIs) were two-sided at a 95% confidence level.

## Results

We used the TwoSampleMR package (version 0.5.6) to extract instrumental SNPs for AD. There were 11,059,641 autosomal SNP-AD associations in the EAGLE consortium. After LD clumping, we identified 12 SNPs that were robustly and independently associated with AD (*p* < 5 × 10^–8^). These 12 SNPs had F-statistics ranging from 14.69 to 61.65, suggesting that there was limited evidence of weak instrument bias. Information on the instrumental variables and their associations with each outcome is presented in Supplementary Table S2.

**FIGURE 1:**
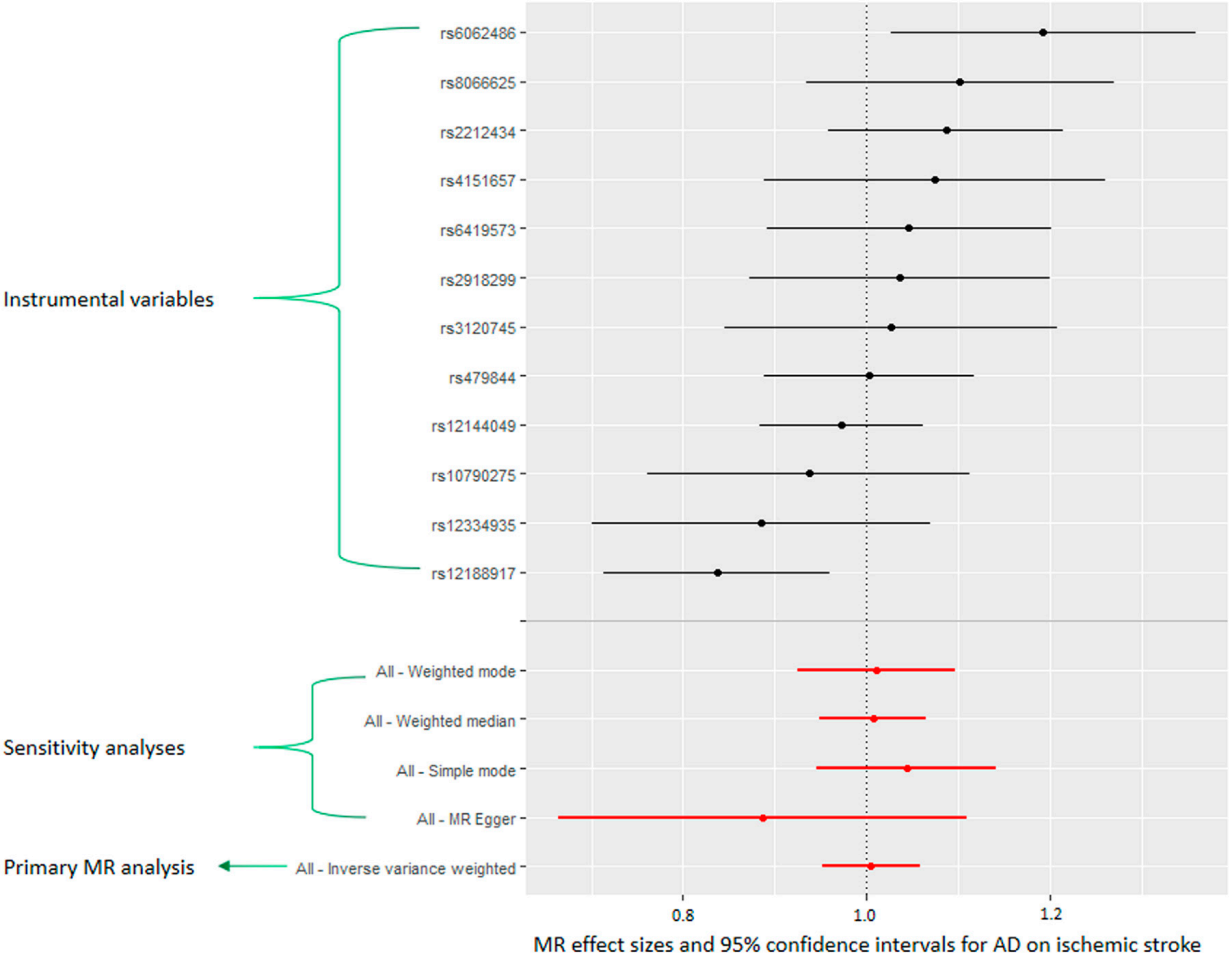
Meta-analytic Mendelian randomization (MR) effect estimates (odds ratios) and 95% confidence intervals for the association between atopic dermatitis (AD) and ischemic stroke. The inverse variance weighted (IVW) method was used in the primary MR analysis. In addition, we performed sensitivity analyses using MR-Egger, weighted median, weighted mode, and simple mode methods.

The results of the main MR analyses are shown in Table 1; Figure 1. There was no causal association of genetically predicted AD with ischemic stroke [odds ratio (OR) = 1.00, 95% confidence interval [CI]: 0.95–1.06, *p* = 0.854], large artery stroke (OR = 1.02, 95% CI: 0.88–1.17, *p* = 0.818), cardioembolic stroke (OR = 1.06, 95% CI: 0.94–1.18, *p* = 0.345), small vessel stroke (OR = 1.05, 95% CI: 0.94–1.17, *p* = 0.418), coronary heart disease (OR = 1.00, 95% CI: 0.94–1.05, *p* = 0.902), and myocardial infarction (OR = 1.03, 95% CI: 0.98–1.09, *p* = 0.188). Consistent with findings from the main MR, sensitivity analyses using MR-Egger, weighted median, simple mode, and weighted mode methods did not reveal any association between AD and any of the outcomes (Table 1; Figure 1). MR-Egger intercepts provided no evidence of directional pleiotropy (all *p* values > 0.10; Table 1). The MR-PRESSO analysis did not indicate any outlier instruments for any of the estimates. In addition, the leave-one-out analysis showed no influential instruments driving the associations, suggesting robust results for all outcomes (Supplementary Table S3). The primary MR analysis had >90% power to detect an OR of 1.20 for the association of AD with ischemic stroke or coronary heart disease at a significance level of 0.05.

**TABLE 1:** Mendelian randomization for the association of atopic dermatitis with ischemic stroke and coronary heart disease.

Outcomes	Association				Heterogeneity		Pleiotropy	
	Method	OR	95% CI	*p*	Q statistic	*p*	Intercept	*p*
Ischemic stroke	IVW (fixed effects)	1.00	0.95–1.06	0.854	18.57	0.069	—	—
	MR-Egger	0.89	0.71–1.12	0.344	16.67	0.082	0.016	0.311
	Weighted median	1.01	0.95–1.07	0.816	—	—	—	—
	Simple mode	1.05	0.95–1.15	0.399	—	—	—	—
	Weighted mode	1.01	0.92–1.10	0.816	—	—	—	—
Ischemic stroke (cardioembolic)	IVW (multiplicative random effects)	1.06	0.94–1.18	0.345	20.48	0.035	—	—
	MR-Egger	0.88	0.54–1.45	0.632	19.44	0.039	0.025	0.482
	Weighted median	0.98	0.87–1.11	0.783	—	—	—	—
	Simple mode	0.98	0.77–1.25	0.897	—	—	—	—
	Weighted mode	0.97	0.83–1.12	0.663	—	—	—	—
Ischemic stroke (large-artery atherosclerosis)	IVW (multiplicative random effects)	1.02	0.88–1.17	0.818	23.42	0.010	—	—
	MR-Egger	1.14	0.60–2.16	0.694	23.11	0.015	−0.016	0.724
	Weighted median	1.04	0.91–1.20	0.536	—	—	—	—
	Simple mode	1.10	0.87–1.39	0.464	—	—	—	—
	Weighted mode	1.06	0.88–1.27	0.553	—	—	—	—
Ischemic stroke (small-vessel)	IVW (fixed effects)	1.05	0.94–1.17	0.418	9.04	0.618	—	—
	MR-Egger	1.12	0.70–1.79	0.649	8.96	0.536	−0.009	0.780
	Weighted median	1.10	0.94–1.27	0.230	—	—	—	—
	Simple mode	1.11	0.88–1.40	0.383	—	—	—	—
	Weighted mode	1.12	0.92–1.37	0.276	—	—	—	—
Coronary heart disease	IVW (multiplicative random effects)	1.00	0.94–1.05	0.902	24.18	0.007	—	—
	MR-Egger	1.00	0.79–1.26	0.998	24.18	0.004	−0.001	0.975
	Weighted median	1.00	0.95–1.05	0.963	—	—	—	—
	Simple mode	1.00	0.91–1.08	0.841	—	—	—	—
	Weighted mode	1.00	0.93–1.07	0.949	—	—	—	—
Myocardial infarction	IVW (fixed effects)	1.03	0.98–1.09	0.188	7.99	0.714	—	—
	MR-Egger	0.89	0.72–1.10	0.310	5.97	0.818	0.021	0.186
	Weighted median	1.01	0.95–1.09	0.698	—	—	—	—
	Simple mode	1.01	0.90–1.13	0.918	—	—	—	—
	Weighted mode	0.99	0.89–1.10	0.850	—	—	—	—

CI, confidence interval; IVW, inverse variance weighted; OR, odds ratio; SE, standard error.

We performed multivariable MR analysis to adjust for asthma and common risk factors of ischemic stroke and coronary heart disease which included type 2 diabetes, hypertension, body mass index, smoking initiation, and alcohol intake frequency. The results of the multivariable MR analysis did not show any association of genetically predicted AD with any of the outcomes (Table 2), supporting the main MR results.

**TABLE 2:** Multivariable MR for assessing the association of atopic dermatitis with ischemic stroke and coronary heart disease.

Outcome	Adjustment for hypertension		Adjustment for T2DM		Adjustment for BMI		Adjustment for alcohol intake frequency		Adjustment for smoking initiation		Adjustment for asthma	
	OR	95% CI	OR	95% CI	OR	95% CI	OR	95% CI	OR	95% CI	OR	95% CI
Ischemic stroke	0.96	0.90–1.02	1.05	0.97–1.12	1.01	0.97–1.05	1.00	0.95–1.05	1.00	0.95–1.05	1.02	0.96–1.07
Ischemic stroke (large-artery atherosclerosis)	1.00	0.88–1.13	1.17	0.99–1.39	1.00	0.91–1.11	1.04	0.94–1.16	1.02	0.93–1.13	1.03	0.88–1.21
Ischemic stroke (cardioembolic)	0.97	0.87–1.08	1.04	0.90–1.19	0.93	0.87–1.01	1.00	0.92–1.09	1.05	0.96–1.14	1.05	0.94–1.18
Ischemic stroke (small-vessel)	0.98	0.86–1.12	1.09	0.92–1.27	1.08	0.97–1.20	1.07	0.96–1.20	1.01	0.90–1.14	1.01	0.92–1.12
Coronary heart disease	1.00	0.89–1.12	0.98	0.87–1.11	0.97	0.92–1.01	1.00	0.93–1.07	1.01	0.96–1.06	0.98	0.93–1.03
Myocardial infarction	1.02	0.93–1.12	0.99	0.86–1.13	0.99	0.94–1.04	1.03	0.95–1.13	1.04	0.99–1.10	1.03	0.99–1.07

T2DM, type 2 diabetes; BMI, body mass index; CI, confidence interval; OR, odds ratio; MR, Mendelian randomization.

We further evaluated the association by extracting instrumental SNPs from an AD data set of the FinnGen study (7,024 cases and 198,740 controls) in the MR-Base database. AD was diagnosed by self-report. Supplementary Table S4 shows the instrumental SNPs. Both the primary MR analysis and sensitivity analysis demonstrated no causal association of AD with ischemic stroke and coronary heart disease (Supplementary Table S5). In addition, we extracted instrumental SNPs from an AD data set of BioBank Japan (https://pheweb.jp/), including 4,296 cases and 163,807 controls (Sakaue et al., 2021). The corresponding data for ischemic stroke (17,671 cases and 192,383 controls) and coronary heart disease (29,319 cases and 183,134) were also obtained from BioBank Japan. AD, ischemic stroke, and coronary heart disease were diagnosed by physicians at each cooperating institution (Nagai et al., 2017). Supplementary Table S6 shows the instrumental SNPs for AD from BioBank Japan. Both the primary MR analysis and sensitivity analysis demonstrated no causal association of AD with ischemic stroke and coronary heart disease (Supplementary Table S7).

Finally, we selected independent instrumental SNPs associated with AD at *p* < 5 × 10^–6^ from the EAGLE consortium (Supplementary Table S8). The primary MR analyses and sensitivity analyses using these instrumental SNPs did not reveal a causal association of AD with ischemic stroke and coronary heart disease (Supplementary Table S9).

## Discussion

In this study, we conducted both univariable and multivariable MR analyses to evaluate possible causal associations of genetically predicted AD with ischemic stroke and coronary heart disease. Our results demonstrated that AD was not causally associated with ischemic stroke, large artery stroke, cardioembolic stroke, small vessel stroke, coronary heart disease, and myocardial infarction. Sensitivity analyses using several reliable methods provided evidence that the MR results were unlikely biased by pleiotropy.

AD is the most common chronic skin inflammatory disease, detrimentally affecting patients’ quality of life. Although it is known that AD is accompanied by allergic comorbidities such as asthma, it is not clear if AD is associated with ischemic stroke and coronary heart disease (Davis et al., 2022). Some authors have speculated that AD-related chronic inflammation might contribute to atherosclerosis development and increase the risk for ischemic stroke and coronary heart disease. In an Asian retrospective cohort study which included 20,323 patients with AD and 20,323 comorbidity-matched subjects, Su et al. (2014) reported an increased risk of ischemic stroke among AD patients (hazard ratio of 1.33). This study employed a population-based design and a large sample size, but it had a shortcoming of misdiagnosis and lacked controlling for traditional risk factors such as diabetes, alcohol use, and cigarette smoking. Another Asian study using the Korean National Health Insurance Data demonstrated that AD cases had elevated stroke and myocardial infarction risk than those without AD, but there were no adjustments for covariates such as cigarette smoking and hypertension (Jung et al., 2021). In addition to these Asian studies, some epidemiology data from other populations also showed positive associations of AD with ischemic stroke and coronary heart disease. For instance, the National Health Interview Surveys of US adults (2010–2012) and a nationwide, register-based, case–control study from Sweden reported that AD was positively associated with ischemic stroke and coronary heart disease (Silverberg, 2015; Ivert et al., 2019). The National Health Interview Survey study used an unspecific questionnaire for diagnosing myocardial infarction; it adjusted for atopic comorbidities but did not control for hypertension and diabetes (Silverberg, 2015). The Swedish study decreased the bias of AD misclassification by using reliable diagnosing methods and had substantial statistical power, but it did not control for obesity and cigarette smoking in the overall analysis (Ivert et al., 2019).

In contrast to these results, in a US propensity-matched case–control study that included 622 patients with AD and 4,263 controls, Marshall et al. (2016) did not detect an independent increased risk of cardiovascular diseases such as ischemic stroke and coronary heart disease among AD cases, after controlling for covariates such as diabetes. In another US study using a cross-sectional design, Drucker et al. (2017) revealed no association of AD with nonfatal myocardial infarction and stroke after adjusting for traditional risk factors including BMI, alcohol intake, and cigarette smoking. Null association was also reported in a German cohort (Standl et al., 2017). These studies pointed out that it was of importance to adjust for covariates, or the analyses may be biased toward false positivity. Indeed, accumulating evidence demonstrates that some important risk factors for stroke and coronary heart disease such as cigarette smoking, alcohol use, and diabetes are more prevalent among AD cases than among controls (Su et al., 2014; Silverberg, 2015; Ali et al., 2018). These risk factors may mediate the potential relationship of AD with ischemic stroke and coronary heart disease. Therefore, without adjustment for these risk factors, the association of AD with ischemic stroke and coronary heart diseases identified in observational studies could not be reliable.

MR is a causal inference technique that uses genetic variants as instruments for causal inference. It can significantly reduce biases of exposure measurement error, reverse causation, and confounding, which are concerns of observational studies evaluating the relationship of AD with ischemic stroke and coronary heart disease. The strengths of this study include the use of a two-sample MR design and high-quality GWAS summary statistics based on a large sample size. In addition, multivariable MR analyses were employed to control for asthma and traditional risk factors (type 2 diabetes, hypertension, BMI, smoking initiation, and alcohol intake frequency). The multivariable MR analyses provided consistent results with those of the main MR effect estimates using the IVW method. Furthermore, the robustness of our results was evaluated by applying detailed sensitivity analyses; the likelihood of horizontal pleiotropy was minimized. Our findings are in line with those of the observational studies by Marshall et al. (2016), Standl et al. (2017), and Drucker et al. (2017) and with a large-scale meta-analysis by Thyssen et al. (2018) which showed that AD was not an independent and clinically relevant risk factor for stroke and myocardial infarction.

Several limitations of our MR study should be considered. Firstly, we were unable to perform stratification by AD severity since the individual-level data were not available. The Swedish study by Ivert et al. (2019) categorized AD cases into non-severe and severe subgroups, evaluating their associations with ischemic stroke and myocardial infarction, respectively. They found that patients with severe AD had a significant association with ischemic stroke, although misclassification of AD severity was present. By contrast, the Korean study by Jung et al. (2021) using the National Health Insurance Data did not find any association between AD severity with stroke and myocardial infarction. In a Danish nationwide cohort study, Andersen et al. (2016) found that patients with mild AD had decreased risk of cardiovascular adverse events. Heterogeneity in sample size, the definition of AD severity, and the characteristics of study participants may be the major reason for these conflicting results. Future MR research may provide additional insights if stratification by AD severity can be performed. Secondly, we could not adjust for the use of corticosteroids which is a key element in treating AD, due to no access to individual-level data. Some authors suggested that corticosteroid use may have possible connections to risk factors for coronary heart disease and stroke such as hypertension among AD patients (Pandher et al., 2022). Thirdly, our MR study mainly focused on GWAS summary statistics for European populations. Although we further used an Asian data set for replication analysis, subtypes of ischemic stroke and coronary heart disease were not evaluated due to a lack of data. Thus, our findings were based on individuals of European descent.

In conclusion, our MR study provides no evidence in support of a causal association of genetically predicted AD with ischemic stroke, large artery stroke, cardioembolic stroke, small vessel stroke, coronary heart disease, and myocardial infarction. AD may not be an independent risk factor for these outcomes.

## Data Availability

This study is based on summary-level data that have been made publically available in the MR-Base platform (http://www.mrbase.org/).
