# Supplementary material for: Investigating the association of atopic dermatitis with ischemic stroke and coronary heart disease: A mendelian randomization study
Source: Front Genet. 2022 Aug 30;13:956850. doi: 10.3389/fgene.2022.956850 (PMC9468876; doi:10.3389/fgene.2022.956850)
Supplement: Supplementary file 1 [file Table1.docx]

Supplementary Table S1 Characteristics of data sources.

| Outcome | Consortium | Year | PMID | Author | Sample size | Ancestry |
| --- | --- | --- | --- | --- | --- | --- |
| Atopic dermatitis | EArly Genetics and Life course Epidemiology (23andME excluded) | 2015 | 26482879 | Paternoster | 10788 cases and 30047 controls | European |
| Ischemic stroke | International Stroke Genetics Consortium | 2018 | 29531354 | Malik | 34217 cases and 406111 controls | European |
| Ischemic stroke (cardioembolic) | International Stroke Genetics Consortium | 2018 | 29531354 | Malik | 7193 cases and 406111 controls | European |
| Ischemic stroke (large-artery atherosclerosis) | International Stroke Genetics Consortium | 2018 | 29531354 | Malik | 4373 cases and 406111 controls | European |
| Ischemic stroke (small-vessel) | International Stroke Genetics Consortium | 2018 | 29531354 | Malik | 5386 cases and 192662 controls | European |
| Coronary heart disease | CARDIoGRAMplusC4D and UK Biobank | 2018 | 29212778 | van der Harst | 122733 cases and 424528 controls | European |
| Myocardial infarction | CARDIoGRAMplusC4D | 2015 | 26343387 | Nikpay | 43676 cases and 128199 controls | Mixed |
| Smoking initiation | GWAS and Sequencing Consortium of Alcohol and Nicotine use | 2019 | 30643251 | Liu | 311629 cases and 321173 controls | European |
| Alcohol intake frequency | Medical Research Council Integrative Epidemiology Unit at the University of Bristol | 2018 | NA | Elsworth | 462346 | European |
| Body mass index | Genetic Investigation of ANthropometric Traits Consortium | 2018 | 30124842 | Yengo | 681275 | European |
| Type 2 diabetes | NA | 2018 | 29632382 | Mahajan | 48286 cases and 250671 controls | European |
| Hypertension | Medical Research Council Integrative Epidemiology Unit at the University of Bristol | 2018 | NA | Elsworth | 54358 cases and 408652 controls | European |
| Asthma | UK Biobank | 2018 | NA | Neale lab | 1693 cases and 359501 controls | European |
