# Supplementary material for: Investigating the association of atopic dermatitis with ischemic stroke and coronary heart disease: A mendelian randomization study
Source: Front Genet. 2022 Aug 30;13:956850. doi: 10.3389/fgene.2022.956850 (PMC9468876; doi:10.3389/fgene.2022.956850)
Supplement: Supplementary file 2 [file Table8.docx]

Supplementary Table S8 Characteristics of the instrumental variables (P<5×10^−6^) for atopic dermatitis and their relationship with cardiovascular diseases.

| Exposure-Outcome | Position | Chr | SNP | Effect allele | Other allele | EAF | Exposure effect |  |  |  | Outcome effect |  |  |
| --- | --- | --- | --- | --- | --- | --- | --- | --- | --- | --- | --- | --- | --- |
|  |  |  |  |  |  |  | β | SE | *P* |  | β | SE | *P* |
| Atopic dermatitis-Ischemic stroke | 68665940 | 12 | rs1038165 | T | C | 0.562 | 0.079 | 0.017 | 3.04E-06 |  | 0.007 | 0.008 | 0.434 |
|  | 118745884 | 11 | rs10790275 | C | G | 0.751 | 0.122 | 0.022 | 2.16E-08 |  | -0.008 | 0.011 | 0.483 |
|  | 1194038 | 17 | rs111375762 | G | T | 0.043 | 0.264 | 0.053 | 7.58E-07 |  | -0.034 | 0.025 | 0.178 |
|  | 35798900 | 14 | rs11156881 | T | C | 0.239 | -0.109 | 0.022 | 7.39E-07 |  | -0.001 | 0.011 | 0.913 |
|  | 6122382 | 10 | rs11256611 | A | C | 0.125 | 0.114 | 0.025 | 4.34E-06 |  | 0.005 | 0.012 | 0.700 |
|  | 8507773 | 1 | rs11581328 | A | G | 0.208 | -0.117 | 0.023 | 3.56E-07 |  | -0.024 | 0.011 | 0.031 |
|  | 118458247 | 5 | rs11738721 | A | G | 0.422 | -0.095 | 0.018 | 1.02E-07 |  | 0.001 | 0.010 | 0.956 |
|  | 152440910 | 1 | rs12144049 | T | C | 0.678 | -0.202 | 0.019 | 2.80E-27 |  | 0.005 | 0.009 | 0.560 |
|  | 131991085 | 5 | rs12188917 | C | T | 0.205 | 0.170 | 0.022 | 2.89E-15 |  | -0.028 | 0.011 | 0.010 |
|  | 126617990 | 8 | rs12334935 | A | G | 0.474 | 0.093 | 0.017 | 4.18E-08 |  | -0.011 | 0.009 | 0.226 |
|  | 123240619 | 4 | rs13152362 | A | G | 0.206 | -0.117 | 0.021 | 5.45E-08 |  | -0.013 | 0.011 | 0.236 |
|  | 81256181 | 8 | rs13266315 | A | G | 0.580 | -0.083 | 0.017 | 1.09E-06 |  | 0.002 | 0.009 | 0.803 |
|  | 14639380 | 9 | rs13302629 | A | G | 0.251 | -0.099 | 0.022 | 4.75E-06 |  | -0.008 | 0.011 | 0.463 |
|  | 8470660 | 2 | rs13419662 | A | G | 0.303 | -0.094 | 0.019 | 4.53E-07 |  | -0.007 | 0.009 | 0.444 |
|  | 162073270 | 4 | rs144143913 | G | A | 0.015 | 0.453 | 0.093 | 1.18E-06 |  | -0.066 | 0.041 | 0.109 |
|  | 68853649 | 8 | rs145009390 | G | C | 0.022 | 0.328 | 0.068 | 1.37E-06 |  | 0.042 | 0.037 | 0.255 |
|  | 11229589 | 16 | rs2041733 | C | T | 0.547 | -0.080 | 0.017 | 1.92E-06 |  | 0.004 | 0.008 | 0.674 |
|  | 76281593 | 11 | rs2212434 | T | C | 0.451 | 0.129 | 0.017 | 2.09E-14 |  | 0.011 | 0.008 | 0.179 |
|  | 36368153 | 11 | rs2433192 | A | G | 0.519 | -0.087 | 0.017 | 2.69E-07 |  | 0.028 | 0.009 | 0.002 |
|  | 53101780 | 3 | rs2581790 | C | T | 0.323 | 0.087 | 0.018 | 1.42E-06 |  | -0.014 | 0.009 | 0.122 |
|  | 35072430 | 2 | rs280729 | G | A | 0.182 | 0.105 | 0.022 | 1.38E-06 |  | 0.013 | 0.0112 | 0.255 |
|  | 8787273 | 19 | rs2918299 | T | C | 0.166 | 0.143 | 0.023 | 5.45E-10 |  | 0.005 | 0.012 | 0.661 |
|  | 153019258 | 1 | rs3120745 | G | A | 0.691 | -0.107 | 0.020 | 4.73E-08 |  | -0.003 | 0.010 | 0.779 |
|  | 31917540 | 6 | rs4151657 | C | T | 0.337 | 0.102 | 0.018 | 7.86E-09 |  | 0.008 | 0.010 | 0.433 |
|  | 65559266 | 11 | rs479844 | G | A | 0.548 | 0.144 | 0.017 | 3.45E-17 |  | 0.001 | 0.008 | 0.956 |
|  | 68634263 | 12 | rs4913279 | C | T | 0.631 | 0.082 | 0.017 | 2.94E-06 |  | 0.015 | 0.009 | 0.105 |
|  | 176766177 | 5 | rs4976685 | G | A | 0.335 | 0.084 | 0.018 | 3.97E-06 |  | -0.004 | 0.009 | 0.704 |
|  | 62302539 | 20 | rs6062486 | A | G | 0.648 | 0.105 | 0.019 | 2.40E-08 |  | 0.020 | 0.009 | 0.023 |
|  | 63520006 | 10 | rs61850526 | T | C | 0.030 | 0.274 | 0.055 | 6.73E-07 |  | -0.028 | 0.022 | 0.199 |
|  | 103027103 | 2 | rs6419573 | C | T | 0.709 | -0.124 | 0.020 | 2.92E-10 |  | -0.006 | 0.010 | 0.551 |
|  | 141491985 | 5 | rs7700687 | T | C | 0.584 | 0.090 | 0.018 | 3.74E-07 |  | -0.011 | 0.009 | 0.189 |
| Atopic dermatitis-Coronary heart disease | 68665940 | 12 | rs1038165 | T | C | 0.562 | 0.079 | 0.017 | 3.04E-06 |  | 0.002 | 0.007 | 0.80 |
|  | 1194038 | 17 | rs111375762 | G | T | 0.043 | 0.264 | 0.053 | 7.58E-07 |  | -0.008 | 0.017 | 0.65 |
|  | 6122382 | 10 | rs11256611 | A | C | 0.125 | 0.114 | 0.025 | 4.34E-06 |  | -0.002 | 0.010 | 0.85 |
|  | 8507773 | 1 | rs11581328 | A | G | 0.208 | -0.117 | 0.023 | 3.56E-07 |  | -0.015 | 0.009 | 0.079 |
|  | 118464075 | 5 | rs11738721 | A | G | 0.422 | -0.095 | 0.018 | 1.02E-07 |  | -0.001 | 0.007 | 0.84 |
|  | 152440910 | 1 | rs12144049 | T | C | 0.678 | -0.202 | 0.019 | 2.80E-27 |  | 0.004 | 0.008 | 0.58 |
|  | 131992809 | 5 | rs12188917 | C | T | 0.205 | 0.170 | 0.022 | 2.89E-15 |  | 0.006 | 0.009 | 0.53 |
|  | 126617990 | 8 | rs12334935 | A | G | 0.474 | 0.093 | 0.017 | 4.18E-08 |  | -0.018 | 0.007 | 0.01 |
|  | 123240619 | 4 | rs13152362 | A | G | 0.206 | -0.117 | 0.021 | 5.45E-08 |  | -0.006 | 0.008 | 0.45 |
|  | 81256181 | 8 | rs13266315 | A | G | 0.580 | -0.083 | 0.017 | 1.09E-06 |  | 0.005 | 0.007 | 0.44 |
|  | 14639380 | 9 | rs13302629 | A | G | 0.251 | -0.099 | 0.022 | 4.75E-06 |  | 0.004 | 0.009 | 0.63 |
|  | 8470660 | 2 | rs13419662 | A | G | 0.303 | -0.094 | 0.019 | 4.53E-07 |  | 0.003 | 0.008 | 0.75 |
|  | 162073270 | 4 | rs144143913 | G | A | 0.015 | 0.453 | 0.093 | 1.18E-06 |  | 0.008 | 0.031 | 0.81 |
|  | 91754695 | 11 | rs1496701 | T | C | 0.015 | 0.750 | 0.162 | 3.91E-06 |  | -0.013 | 0.031 | 0.69 |
|  | 98895472 | 8 | rs188557945 | A | G | 0.015 | 0.632 | 0.137 | 4.29E-06 |  | -0.031 | 0.028 | 0.27 |
|  | 11229589 | 16 | rs2041733 | C | T | 0.547 | -0.080 | 0.017 | 1.92E-06 |  | -0.0001 | 0.007 | 0.99 |
|  | 76281593 | 11 | rs2212434 | T | C | 0.451 | 0.129 | 0.017 | 2.09E-14 |  | 0.003 | 0.007 | 0.71 |
|  | 36368153 | 11 | rs2433192 | A | G | 0.519 | -0.087 | 0.017 | 2.69E-07 |  | -0.006 | 0.007 | 0.36 |
|  | 53101780 | 3 | rs2581790 | C | T | 0.323 | 0.087 | 0.018 | 1.42E-06 |  | 0.012 | 0.007 | 0.12 |
|  | 35072430 | 2 | rs280729 | G | A | 0.182 | 0.105 | 0.022 | 1.38E-06 |  | -0.015 | 0.009 | 0.1 |
|  | 8787273 | 19 | rs2918299 | T | C | 0.166 | 0.143 | 0.023 | 5.45E-10 |  | -0.019 | 0.010 | 0.049 |
|  | 153019258 | 1 | rs3120745 | G | A | 0.691 | -0.107 | 0.020 | 4.73E-08 |  | 0.004 | 0.008 | 0.63 |
|  | 31917540 | 6 | rs4151657 | C | T | 0.337 | 0.102 | 0.018 | 7.86E-09 |  | 0.019 | 0.008 | 0.014 |
|  | 65551957 | 11 | rs479844 | G | A | 0.548 | 0.144 | 0.017 | 3.45E-17 |  | 0.014 | 0.007 | 0.05 |
|  | 68634263 | 12 | rs4913279 | C | T | 0.631 | 0.082 | 0.017 | 2.94E-06 |  | -0.003 | 0.007 | 0.72 |
|  | 176766177 | 5 | rs4976685 | G | A | 0.335 | 0.084 | 0.018 | 3.97E-06 |  | 0.004 | 0.008 | 0.58 |
|  | 62302539 | 20 | rs6062486 | A | G | 0.648 | 0.105 | 0.019 | 2.40E-08 |  | 0.006 | 0.007 | 0.44 |
|  | 63520006 | 10 | rs61850526 | T | C | 0.030 | 0.274 | 0.055 | 6.73E-07 |  | -0.016 | 0.019 | 0.39 |
|  | 103029410 | 2 | rs6419573 | C | T | 0.709 | -0.124 | 0.0197 | 2.92E-10 |  | 0.011 | 0.008 | 0.21 |
|  | 141491985 | 5 | rs7700687 | T | C | 0.584 | 0.090 | 0.018 | 3.74E-07 |  | 0.016 | 0.007 | 0.022 |
|  | 40390629 | 17 | rs8066625 | A | G | 0.107 | 0.176 | 0.032 | 3.84E-08 |  | -0.009 | 0.012 | 0.43 |
|  | 212858797 | 1 | rs906363 | C | T | 0.148 | 0.112 | 0.023 | 1.52E-06 |  | -0.007 | 0.010 | 0.48 |

EAF, effect allele frequency; SNP, single nucleotide polymorphism; SE, standard error.
