# Supplementary material for: Investigating the association of atopic dermatitis with ischemic stroke and coronary heart disease: A mendelian randomization study
Source: Front Genet. 2022 Aug 30;13:956850. doi: 10.3389/fgene.2022.956850 (PMC9468876; doi:10.3389/fgene.2022.956850)
Supplement: Supplementary file 3 [file Table7.docx]

Supplementary Table S7 MR for the association of AD with ischemic stroke and coronary heart disease using exposure and outcome datasets from the BBJ.

| Outcomes | Association | | | |  | Pleiotropy |  |
| --- | --- | --- | --- | --- | --- | --- | --- |
|  | Method | OR | 95% CI | *P* |  | Intercept | *P* |
| Ischemic stroke | IVW | 1.00 | 0.95-1.05 | 0.984 |  | - | - |
|  | MR-Egger | 1.01 | 0.90-1.13 | 0.902 |  | -0.002 | 0.899 |
|  | Weighted median | 1.00 | 0.96-1.04 | 0.950 |  | - | - |
|  | Simple mode | 0.99 | 0.94-1.05 | 0.777 |  | - | - |
| Coronary heart disease | IVW | 0.98 | 0.94-1.01 | 0.142 |  | - | - |
|  | MR-Egger | 0.94 | 0.86-1.03 | 0.181 |  | 0.009 | 0.392 |
|  | Weighted median | 0.96 | 0.92-1.01 | 0.104 |  | - | - |
|  | Simple mode | 0.99 | 0.94-1.04 | 0.667 |  | - | - |

AD, atopic dermatitis; BBJ, BioBank Japan; CI, confidence interval; IVW, inverse variance weighted; OR, odds ratio; SE, standard error.
