# Supplementary figures and images for: Investigating the association of atopic dermatitis with ischemic stroke and coronary heart disease: A mendelian randomization study
Source: Front Genet. 2022 Aug 30;13:956850. doi: 10.3389/fgene.2022.956850 (PMC9468876; doi:10.3389/fgene.2022.956850)

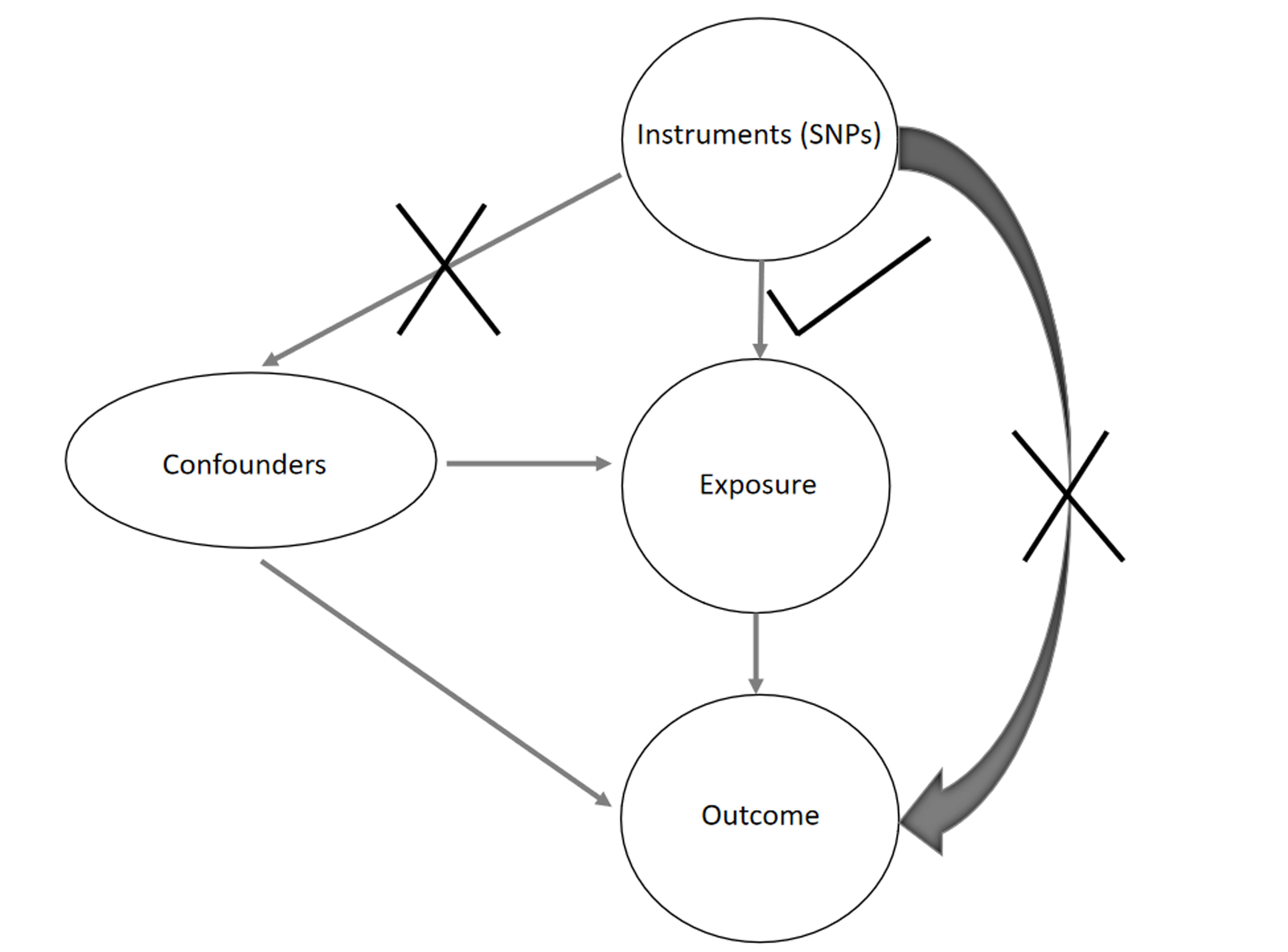

Supplement: Supplementary file 4 [file Image1.tif]
