# Supplementary material for: Investigating the association of atopic dermatitis with ischemic stroke and coronary heart disease: A mendelian randomization study
Source: Front Genet. 2022 Aug 30;13:956850. doi: 10.3389/fgene.2022.956850 (PMC9468876; doi:10.3389/fgene.2022.956850)
Supplement: Supplementary file 5 [file Table9.docx]

Supplementary Table S9 MR for the association of AD with ischemic stroke and coronary heart disease using instrumental SNPs with *P* < 5×10^−6^.

| Outcomes | Association | | | |  | Pleiotropy |  |
| --- | --- | --- | --- | --- | --- | --- | --- |
|  | Method | OR | 95% CI | *P* |  | Intercept | *P* |
| Ischemic stroke | IVW | 1.00 | 0.97-1.04 | 0.873 |  | - | - |
|  | MR-Egger | 0.97 | 0.86-1.09 | 0.585 |  | 0.005 | 0.526 |
|  | Weighted median | 1.01 | 0.96-1.05 | 0.810 |  | - | - |
|  | Simple mode | 1.07 | 0.97-1.17 | 0.210 |  | - | - |
|  | Weighted mode | 1.02 | 0.94-1.11 | 0.681 |  | - | - |
| Coronary heart disease | IVW | 1.00 | 0.97-1.03 | 0.930 |  | - | - |
|  | MR-Egger | 0.96 | 0.91-1.03 | 0.259 |  | 0.005 | 0.225 |
|  | Weighted median | 0.98 | 0.95-1.02 | 0.351 |  | - | - |
|  | Simple mode | 0.97 | 0.91-1.03 | 0.370 |  | - | - |
|  | Weighted mode | 0.98 | 0.93-1.03 | 0.339 |  | - | - |

AD, atopic dermatitis; CI, confidence interval; IVW, inverse variance weighted; OR, odds ratio; SE, standard error; SNP, single nucleotide polymorphism.
