# Supplementary material for: Investigating the association of atopic dermatitis with ischemic stroke and coronary heart disease: A mendelian randomization study
Source: Front Genet. 2022 Aug 30;13:956850. doi: 10.3389/fgene.2022.956850 (PMC9468876; doi:10.3389/fgene.2022.956850)
Supplement: Supplementary file 6 [file Table2.docx]

Supplementary Table S2 Characteristics of the instrumental variables for atopic dermatitis and their relationship with cardiovascular diseases.

| Exposure-Outcome | Position | Chr | SNP | Effect allele | Other allele | EAF | Exposure effect |  |  |  | Outcome effect |  |  |
| --- | --- | --- | --- | --- | --- | --- | --- | --- | --- | --- | --- | --- | --- |
|  |  |  |  |  |  |  | β | SE | *P* |  | β | SE | *P* |
| Atopic dermatitis-Ischemic stroke | 118745884 | 11 | rs10790275 | C | G | 0.751 | 0.122 | 0.022 | 2.160e-08 |  | -0.001 | 0.011 | 0.483 |
|  | 152440910 | 1 | rs12144049 | T | C | 0.678 | -0.202 | 0.019 | 2.800e-27 |  | 0.005 | 0.009 | 0.560 |
|  | 131991085 | 5 | rs12188917 | C | T | 0.205 | 0.170 | 0.021 | 2.890e-15 |  | -0.028 | 0.011 | 0.010 |
|  | 126617990 | 8 | rs12334935 | A | G | 0.474 | 0.093 | 0.017 | 4.180e-08 |  | -0.011 | 0.009 | 0.226 |
|  | 76281593 | 11 | rs2212434 | T | C | 0.451 | 0.129 | 0.017 | 2.090e-14 |  | 0.011 | 0.008 | 0.179 |
|  | 8787273 | 19 | rs2918299 | T | C | 0.166 | 0.143 | 0.023 | 5.450e-10 |  | 0.005 | 0.012 | 0.661 |
|  | 153019258 | 1 | rs3120745 | G | A | 0.691 | -0.107 | 0.020 | 4.730e-08 |  | -0.003 | 0.010 | 0.779 |
|  | 31917540 | 6 | rs4151657 | C | T | 0.337 | 0.102 | 0.018 | 7.860e-09 |  | 0.008 | 0.010 | 0.433 |
|  | 65559266 | 11 | rs479844 | G | A | 0.548 | 0.144 | 0.017 | 3.450e-17 |  | 0.001 | 0.008 | 0.956 |
|  | 62302539 | 20 | rs6062486 | A | G | 0.648 | 0.105 | 0.019 | 2.400e-08 |  | 0.020 | 0.009 | 0.023 |
|  | 103027103 | 2 | rs6419573 | C | T | 0.709 | -0.124 | 0.020 | 2.920e-10 |  | -0.006 | 0.010 | 0.551 |
|  | 40390629 | 17 | rs8066625 | A | G | 0.107 | 0.176 | 0.032 | 3.840e-8 |  | 0.018 | 0.015 | 0.232 |
| Atopic dermatitis-Ischemic stroke (cardioembolic) | 118745884 | 11 | rs10790275 | C | G | 0.751 | 0.122 | 0.022 | 2.160e-08 |  | 0.004 | 0.022 | 0.867 |
|  | 152440910 | 1 | rs12144049 | T | C | 0.678 | -0.202 | 0.019 | 2.800e-27 |  | 0.020 | 0.020 | 0.310 |
|  | 131991085 | 5 | rs12188917 | C | T | 0.205 | 0.170 | 0.021 | 2.890e-15 |  | -0.006 | 0.022 | 0.801 |
|  | 126617990 | 8 | rs12334935 | A | G | 0.474 | 0.093 | 0.017 | 4.180e-08 |  | -0.013 | 0.017 | 0.444 |
|  | 76281593 | 11 | rs2212434 | T | C | 0.451 | 0.129 | 0.017 | 2.090e-14 |  | -0.003 | 0.017 | 0.855 |
|  | 8787273 | 19 | rs2918299 | T | C | 0.166 | 0.143 | 0.023 | 5.450e-10 |  | 0.052 | 0.023 | 0.026 |
|  | 153019258 | 1 | rs3120745 | G | A | 0.691 | -0.107 | 0.020 | 4.730e-08 |  | -0.007 | 0.021 | 0.736 |
|  | 31917540 | 6 | rs4151657 | C | T | 0.337 | 0.102 | 0.018 | 7.860e-09 |  | 0.041 | 0.019 | 0.027 |
|  | 65559266 | 11 | rs479844 | G | A | 0.548 | 0.144 | 0.017 | 3.450e-17 |  | 0.017 | 0.018 | 0.347 |
|  | 62302539 | 20 | rs6062486 | A | G | 0.648 | 0.105 | 0.019 | 2.400e-08 |  | 0.034 | 0.018 | 0.063 |
|  | 103027103 | 2 | rs6419573 | C | T | 0.709 | -0.124 | 0.020 | 2.920e-10 |  | 0.031 | 0.020 | 0.119 |
|  | 40390629 | 17 | rs8066625 | A | G | 0.107 | 0.176 | 0.032 | 3.840e-8 |  | 0.058 | 0.030 | 0.057 |
| Atopic dermatitis-Ischemic stroke (large-artery atherosclerosis) | 118745884 | 11 | rs10790275 | C | G | 0.751 | 0.122 | 0.022 | 2.160e-08 |  | -0.035 | 0.027 | 0.191 |
|  | 152440910 | 1 | rs12144049 | T | C | 0.678 | -0.202 | 0.019 | 2.800e-27 |  | -0.004 | 0.023 | 0.875 |
|  | 131991085 | 5 | rs12188917 | C | T | 0.205 | 0.170 | 0.021 | 2.890e-15 |  | -0.011 | 0.025 | 0.677 |
|  | 126617990 | 8 | rs12334935 | A | G | 0.474 | 0.093 | 0.017 | 4.180e-08 |  | -0.048 | 0.021 | 0.021 |
|  | 76281593 | 11 | rs2212434 | T | C | 0.451 | 0.129 | 0.017 | 2.090e-14 |  | 0.008 | 0.020 | 0.682 |
|  | 8787273 | 19 | rs2918299 | T | C | 0.166 | 0.143 | 0.023 | 5.450e-10 |  | 0.059 | 0.028 | 0.033 |
|  | 153019258 | 1 | rs3120745 | G | A | 0.691 | -0.107 | 0.020 | 4.730e-08 |  | 0.035 | 0.024 | 0.149 |
|  | 31917540 | 6 | rs4151657 | C | T | 0.337 | 0.102 | 0.018 | 7.860e-09 |  | 0.013 | 0.022 | 0.544 |
|  | 65559266 | 11 | rs479844 | G | A | 0.548 | 0.144 | 0.017 | 3.450e-17 |  | 0.019 | 0.021 | 0.375 |
|  | 62302539 | 20 | rs6062486 | A | G | 0.648 | 0.105 | 0.019 | 2.400e-08 |  | 0.017 | 0.022 | 0.433 |
|  | 103027103 | 2 | rs6419573 | C | T | 0.709 | -0.124 | 0.020 | 2.920e-10 |  | -0.048 | 0.022 | 0.028 |
|  | 40390629 | 17 | rs8066625 | A | G | 0.107 | 0.176 | 0.032 | 3.840e-8 |  | -0.067 | 0.039 | 0.082 |
| Atopic dermatitis-Ischemic stroke (small-vessel) | 118745884 | 11 | rs10790275 | C | G | 0.751 | 0.122 | 0.022 | 2.160e-08 |  | -0.047 | 0.029 | 0.106 |
|  | 152440910 | 1 | rs12144049 | T | C | 0.678 | -0.202 | 0.019 | 2.800e-27 |  | -0.029 | 0.026 | 0.251 |
|  | 131991085 | 5 | rs12188917 | C | T | 0.205 | 0.170 | 0.021 | 2.890e-15 |  | -0.013 | 0.029 | 0.669 |
|  | 126617990 | 8 | rs12334935 | A | G | 0.474 | 0.093 | 0.017 | 4.180e-08 |  | -0.022 | 0.023 | 0.335 |
|  | 76281593 | 11 | rs2212434 | T | C | 0.451 | 0.129 | 0.017 | 2.090e-14 |  | 0.020 | 0.023 | 0.370 |
|  | 8787273 | 19 | rs2918299 | T | C | 0.166 | 0.143 | 0.023 | 5.450e-10 |  | -0.016 | 0.032 | 0.632 |
|  | 153019258 | 1 | rs3120745 | G | A | 0.691 | -0.107 | 0.020 | 4.730e-08 |  | -0.003 | 0.026 | 0.906 |
|  | 31917540 | 6 | rs4151657 | C | T | 0.337 | 0.102 | 0.018 | 7.860e-09 |  | 0.034 | 0.026 | 0.200 |
|  | 65559266 | 11 | rs479844 | G | A | 0.548 | 0.144 | 0.017 | 3.450e-17 |  | 0.001 | 0.023 | 0.983 |
|  | 62302539 | 20 | rs6062486 | A | G | 0.648 | 0.105 | 0.019 | 2.400e-08 |  | 0.027 | 0.024 | 0.267 |
|  | 103027103 | 2 | rs6419573 | C | T | 0.709 | -0.124 | 0.020 | 2.920e-10 |  | -0.020 | 0.027 | 0.461 |
|  | 40390629 | 17 | rs8066625 | A | G | 0.107 | 0.176 | 0.032 | 3.840e-8 |  | 0.015 | 0.040 | 0.703 |
| Atopic dermatitis-Coronary heart disease | 152440910 | 1 | rs12144049 | T | C | 0.678 | -0.202 | 0.019 | 2.800e-27 |  | 0.004 | 0.008 | 0.580 |
|  | 131991085 | 5 | rs12188917 | C | T | 0.205 | 0.170 | 0.021 | 2.890e-15 |  | 0.006 | 0.009 | 0.530 |
|  | 126617990 | 8 | rs12334935 | A | G | 0.474 | 0.093 | 0.017 | 4.180e-08 |  | -0.018 | 0.007 | 0.010 |
|  | 76281593 | 11 | rs2212434 | T | C | 0.451 | 0.129 | 0.017 | 2.090e-14 |  | 0.003 | 0.007 | 0.710 |
|  | 8787273 | 19 | rs2918299 | T | C | 0.166 | 0.143 | 0.023 | 5.450e-10 |  | -0.019 | 0.010 | 0.049 |
|  | 153019258 | 1 | rs3120745 | G | A | 0.691 | -0.107 | 0.020 | 4.730e-08 |  | 0.004 | 0.008 | 0.630 |
|  | 31917540 | 6 | rs4151657 | C | T | 0.337 | 0.102 | 0.018 | 7.860e-09 |  | 0.019 | 0.008 | 0.014 |
|  | 65559266 | 11 | rs479844 | G | A | 0.548 | 0.144 | 0.017 | 3.450e-17 |  | 0.014 | 0.007 | 0.050 |
|  | 62302539 | 20 | rs6062486 | A | G | 0.648 | 0.105 | 0.019 | 2.400e-08 |  | 0.006 | 0.007 | 0.440 |
|  | 103027103 | 2 | rs6419573 | C | T | 0.709 | -0.124 | 0.020 | 2.920e-10 |  | 0.011 | 0.008 | 0.210 |
|  | 40390629 | 17 | rs8066625 | A | G | 0.107 | 0.176 | 0.032 | 3.840e-8 |  | -0.009 | 0.012 | 0.430 |
| Atopic dermatitis-Myocardial infarction | 118745884 | 11 | rs10790275 | C | G | 0.751 | 0.122 | 0.022 | 2.160e-08 |  | 0.012 | 0.014 | 0.385 |
|  | 152440910 | 1 | rs12144049 | T | C | 0.678 | -0.202 | 0.019 | 2.800e-27 |  | 0.009 | 0.012 | 0.462 |
|  | 131991085 | 5 | rs12188917 | C | T | 0.205 | 0.170 | 0.021 | 2.890e-15 |  | 0.006 | 0.013 | 0.660 |
|  | 126617990 | 8 | rs12334935 | A | G | 0.474 | 0.093 | 0.017 | 4.180e-08 |  | 0.002 | 0.010 | 0.879 |
|  | 76281593 | 11 | rs2212434 | T | C | 0.451 | 0.129 | 0.017 | 2.090e-14 |  | -0.001 | 0.011 | 0.945 |
|  | 8787273 | 19 | rs2918299 | T | C | 0.166 | 0.143 | 0.023 | 5.450e-10 |  | -0.001 | 0.015 | 0.950 |
|  | 153019258 | 1 | rs3120745 | G | A | 0.691 | -0.107 | 0.020 | 4.730e-08 |  | -0.014 | 0.012 | 0.272 |
|  | 31917540 | 6 | rs4151657 | C | T | 0.337 | 0.102 | 0.018 | 7.860e-09 |  | 0.020 | 0.011 | 0.067 |
|  | 65559266 | 11 | rs479844 | G | A | 0.548 | 0.144 | 0.017 | 3.450e-17 |  | 0.018 | 0.010 | 0.084 |
|  | 62302539 | 20 | rs6062486 | A | G | 0.648 | 0.105 | 0.019 | 2.400e-08 |  | 0.007 | 0.011 | 0.551 |
|  | 103027103 | 2 | rs6419573 | C | T | 0.709 | -0.124 | 0.020 | 2.920e-10 |  | 0.006 | 0.011 | 0.582 |
|  | 40390629 | 17 | rs8066625 | A | G | 0.107 | 0.176 | 0.032 | 3.840e-8 |  | 0.002 | 0.020 | 0.929 |

EAF, effect allele frequency; SNP, single nucleotide polymorphism; SE, standard error.
