# Supplementary material for: Investigating the association of atopic dermatitis with ischemic stroke and coronary heart disease: A mendelian randomization study
Source: Front Genet. 2022 Aug 30;13:956850. doi: 10.3389/fgene.2022.956850 (PMC9468876; doi:10.3389/fgene.2022.956850)
Supplement: Supplementary file 7 [file Table5.docx]

Supplementary Table S5 MR for the association of atopic dermatitis with ischemic stroke and coronary heart disease using exposure dataset from the FinnGen study.

| Outcomes | Association | | | |  | Pleiotropy |  |
| --- | --- | --- | --- | --- | --- | --- | --- |
|  | Method | OR | 95% CI | *P* |  | Intercept | *P* |
| Ischemic stroke (all) | IVW | 1.03 | 0.99-1.06 | 0.128 |  | - | - |
|  | MR-Egger | 1.10 | 1.00-1.21 | 0.068 |  | -0.011 | 0.148 |
|  | Weighted median | 1.03 | 0.99-1.08 | 0.158 |  | - | - |
|  | Simple mode | 1.04 | 0.98-1.12 | 0.382 |  | - | - |
|  | Weighted mode | 1.05 | 0.98-1.12 | 0.195 |  | - | - |
| Ischemic stroke (cardioembolic) | IVW | 1.01 | 0.92-1.11 | 0.888 |  | - | - |
|  | MR-Egger | 1.23 | 1.00-1.50 | 0.068 |  | -0.036 | 0.053 |
|  | Weighted median | 1.02 | 0.91-1.13 | 0.764 |  | - | - |
|  | Simple mode | 1.08 | 0.86-1.35 | 0.541 |  | - | - |
|  | Weighted mode | 1.03 | 0.84-1.28 | 0.760 |  | - | - |
| Ischemic stroke (large-artery atherosclerosis) | IVW | 1.07 | 0.98-1.17 | 0.117 |  | - | - |
|  | MR-Egger | 1.17 | 0.95-1.45 | 0.170 |  | -0.016 | 0.386 |
|  | Weighted median | 1.03 | 0.92-1.16 | 0.610 |  | - | - |
|  | Simple mode | 1.00 | 0.83-1.21 | 0.889 |  | - | - |
|  | Weighted mode | 1.03 | 0.87-1.22 | 0.743 |  | - | - |
| Ischemic stroke (small-vessel) | IVW | 1.05 | 0.96-1.16 | 0.274 |  | - | - |
|  | MR-Egger | 1.08 | 0.87-1.34 | 0.491 |  | -0.005 | 0.799 |
|  | Weighted median | 1.05 | 0.93-1.20 | 0.415 |  | - | - |
|  | Simple mode | 0.92 | 0.73-1.14 | 0.450 |  | - | - |
|  | Weighted mode | 1.07 | 0.91-1.26 | 0.415 |  | - | - |
| Coronary heart disease | IVW | 1.02 | 0.96-1.08 | 0.502 |  | - | - |
|  | MR-Egger | 0.98 | 0.88-1.09 | 0.055 |  | 0.009 | 0.380 |
|  | Weighted median | 1.00 | 0.96-1.03 | 0.849 |  | - | - |
|  | Simple mode | 1.00 | 0.94-1.05 | 0.874 |  | - | - |
|  | Weighted mode | 0.99 | 0.95-1.04 | 0.783 |  | - | - |
| Myocardial infarction | IVW | 1.02 | 0.97-1.07 | 0.496 |  | - | - |
|  | MR-Egger | 0.99 | 0.88-1.12 | 0.913 |  | 0.005 | 0.659 |
|  | Weighted median | 1.00 | 0.94-1.06 | 0.872 |  | - | - |
|  | Simple mode | 0.99 | 0.90-1.08 | 0.827 |  | - | - |
|  | Weighted mode | 0.97 | 0.90-1.06 | 0.550 |  | - | - |

AD, atopic dermatitis; CI: confidence interval; IVW, inverse variance weighted; MR, Mendelian randomization; OR: odds ratio; SE, standard error.
