# Supplementary material for: Investigating the association of atopic dermatitis with ischemic stroke and coronary heart disease: A mendelian randomization study
Source: Front Genet. 2022 Aug 30;13:956850. doi: 10.3389/fgene.2022.956850 (PMC9468876; doi:10.3389/fgene.2022.956850)
Supplement: Supplementary file 8 [file Table3.docx]

Supplementary Table S3 Leave-one-out analyses using the IVW method.

| Outcome | SNP | OR | 95% CI |
| --- | --- | --- | --- |
| Ischemic stroke | All | 1.00 | 0.95-1.06 |
|  | Removing rs10790275 | 1.01 | 0.95-1.07 |
|  | Removing rs12144049 | 1.01 | 0.95-1.08 |
|  | Removing rs12188917 | 1.03 | 0.98-1.07 |
|  | Removing rs12334935 | 1.01 | 0.96-1.07 |
|  | Removing rs2212434 | 1.00 | 0.94-1.05 |
|  | Removing rs2918299 | 1.00 | 0.95-1.06 |
|  | Removing rs3120745 | 1.00 | 0.95-1.06 |
|  | Removing rs4151657 | 1.00 | 0.95-1.06 |
|  | Removing rs479844 | 1.01 | 0.95-1.07 |
|  | Removing rs6062486 | 0.99 | 0.95-1.04 |
|  | Removing rs6419573 | 1.00 | 0.95-1.06 |
|  | Removing rs8066625 | 1.00 | 0.95-1.06 |
| Ischemic stroke (cardioembolic) | All | 1.06 | 0.94-1.18 |
|  | Removing rs10790275 | 1.06 | 0.94-1.20 |
|  | Removing rs12144049 | 1.10 | 0.97-1.24 |
|  | Removing rs12188917 | 1.07 | 0.94-1.21 |
|  | Removing rs12334935 | 1.07 | 0.95-1.20 |
|  | Removing rs2212434 | 1.07 | 0.94-1.21 |
|  | Removing rs2918299 | 1.03 | 0.92-1.16 |
|  | Removing rs3120745 | 1.06 | 0.93-1.19 |
|  | Removing rs4151657 | 1.04 | 0.93-1.16 |
|  | Removing rs479844 | 1.05 | 0.92-1.19 |
|  | Removing rs6062486 | 1.04 | 0.93-1.17 |
|  | Removing rs6419573 | 1.08 | 0.97-1.21 |
|  | Removing rs8066625 | 1.04 | 0.92-1.16 |
| Ischemic stroke (large-artery atherosclerosis) | All | 1.02 | 0.88-1.17 |
|  | Removing rs10790275 | 1.03 | 0.89-1.20 |
|  | Removing rs12144049 | 1.02 | 0.86-1.20 |
|  | Removing rs12188917 | 1.03 | 0.88-1.20 |
|  | Removing rs12334935 | 1.05 | 0.92-1.19 |
|  | Removing rs2212434 | 1.01 | 0.86-1.18 |
|  | Removing rs2918299 | 0.99 | 0.86-1.14 |
|  | Removing rs3120745 | 1.04 | 0.89-1.20 |
|  | Removing rs4151657 | 1.01 | 0.87-1.18 |
|  | Removing rs479844 | 1.00 | 0.86-1.17 |
|  | Removing rs6062486 | 1.01 | 0.86-1.17 |
|  | Removing rs6419573 | 0.98 | 0.86-1.13 |
|  | Removing rs8066625 | 1.04 | 0.90-1.20 |
| Ischemic stroke (small-vessel) | All | 1.05 | 0.94-1.17 |
|  | Removing rs10790275 | 1.07 | 0.96-1.20 |
|  | Removing rs12144049 | 1.02 | 0.90-1.16 |
|  | Removing rs12188917 | 1.06 | 0.94-1.19 |
|  | Removing rs12334935 | 1.06 | 0.95-1.19 |
|  | Removing rs2212434 | 1.03 | 0.92-1.16 |
|  | Removing rs2918299 | 1.06 | 0.94-1.19 |
|  | Removing rs3120745 | 1.05 | 0.93-1.17 |
|  | Removing rs4151657 | 1.03 | 0.92-1.16 |
|  | Removing rs479844 | 1.05 | 0.94-1.19 |
|  | Removing rs6062486 | 1.03 | 0.92-1.16 |
|  | Removing rs6419573 | 1.04 | 0.93-1.16 |
|  | Removing rs8066625 | 1.04 | 0.93-1.17 |
| Coronary heart disease | All | 1.00 | 0.94-1.05 |
|  | Removing rs12144049 | 1.00 | 0.94-1.07 |
|  | Removing rs12188917 | 0.99 | 0.93-1.05 |
|  | Removing rs12334935 | 1.01 | 0.96-1.06 |
|  | Removing rs2212434 | 0.99 | 0.94-1.06 |
|  | Removing rs2918299 | 1.01 | 0.95-1.06 |
|  | Removing rs3120745 | 1.00 | 0.94-1.06 |
|  | Removing rs4151657 | 0.99 | 0.94-1.04 |
|  | Removing rs479844 | 0.98 | 0.93-1.04 |
|  | Removing rs6062486 | 0.99 | 0.94-1.05 |
|  | Removing rs6419573 | 1.00 | 0.95-1.06 |
|  | Removing rs8066625 | 1.00 | 0.94-1.06 |
| Myocardial infarction | All | 1.03 | 0.98-1.09 |
|  | Removing rs10790275 | 1.03 | 0.98-1.09 |
|  | Removing rs12144049 | 1.05 | 1.00-1.12 (*P*=0.065) |
|  | Removing rs12188917 | 1.03 | 0.98-1.09 |
|  | Removing rs12334935 | 1.04 | 0.98-1.09 |
|  | Removing rs2212434 | 1.04 | 0.98-1.10 |
|  | Removing rs2918299 | 1.04 | 0.98-1.09 |
|  | Removing rs3120745 | 1.03 | 0.98-1.08 |
|  | Removing rs4151657 | 1.02 | 0.97-1.08 |
|  | Removing rs479844 | 1.02 | 0.97-1.08 |
|  | Removing rs6062486 | 1.03 | 0.98-1.09 |
|  | Removing rs6419573 | 1.04 | 0.99-1.10 |
|  | Removing rs8066625 | 1.04 | 0.98-1.09 |

IVW, inverse variance weighted.
