# Supplementary material for: Investigating the association of atopic dermatitis with ischemic stroke and coronary heart disease: A mendelian randomization study
Source: Front Genet. 2022 Aug 30;13:956850. doi: 10.3389/fgene.2022.956850 (PMC9468876; doi:10.3389/fgene.2022.956850)
Supplement: Supplementary file 9 [file Table4.docx]

Supplementary Table S4 Instrumental SNPs for atopic dermatitis from the FinnGen study.

| SNP | Chr | Position | Effect allele | Other allele | Exposure effect | | |
| --- | --- | --- | --- | --- | --- | --- | --- |
|  |  |  |  |  | β | SE | *P* |
| rs10208309 | 2 | 61178509 | G | C | 0.117 | 0.021 | 2.74E-08 |
| rs11236814 | 11 | 76343428 | T | A | -0.197 | 0.029 | 4.78E-12 |
| rs12731336 | 1 | 152448098 | G | A | -0.299 | 0.044 | 1.43E-11 |
| rs2227472 | 12 | 68649133 | C | T | 0.117 | 0.018 | 1.77E-10 |
| rs2236506 | 20 | 62321655 | A | G | 0.185 | 0.023 | 1.72E-15 |
| rs28371176 | 6 | 32612839 | G | A | -0.250 | 0.044 | 1.28E-08 |
| rs34666276 | 17 | 47379486 | C | T | 0.135 | 0.018 | 1.35E-13 |
| rs58453446 | 6 | 90948093 | C | G | -0.123 | 0.021 | 3.80E-09 |
| rs61814899 | 1 | 152069131 | A | G | 0.680 | 0.108 | 2.65E-10 |
| rs61839660 | 10 | 6094697 | T | C | 0.269 | 0.047 | 8.59E-09 |
| rs6534340 | 4 | 123037548 | C | A | -0.126 | 0.020 | 1.49E-10 |
| rs6543132 | 2 | 103029410 | G | A | -0.140 | 0.023 | 1.51E-09 |
| rs6991991 | 8 | 81271432 | C | T | -0.109 | 0.019 | 2.22E-08 |
| rs77531520 | 11 | 36432445 | T | C | 0.240 | 0.036 | 2.64E-11 |
| rs7936070 | 11 | 76293527 | T | G | 0.159 | 0.018 | 5.05E-18 |
| rs8108687 | 19 | 927971 | G | A | 0.112 | 0.020 | 4.20E-08 |
| rs847 | 5 | 131996669 | C | T | -0.122 | 0.019 | 1.10E-10 |

SNP, single nucleotide polymorphism; SE, standard error.
