# Supplementary material for: Investigating the association of atopic dermatitis with ischemic stroke and coronary heart disease: A mendelian randomization study
Source: Front Genet. 2022 Aug 30;13:956850. doi: 10.3389/fgene.2022.956850 (PMC9468876; doi:10.3389/fgene.2022.956850)
Supplement: Supplementary file 10 [file Table6.docx]

Supplementary Table S6 Instrumental SNPs for atopic dermatitis from the BioBank Japan.

| SNP | Chr | Position | Effect allele | Other allele | Exposure effect | | |
| --- | --- | --- | --- | --- | --- | --- | --- |
|  |  |  |  |  | β | SE | *P* |
| rs59039403 | 11 | 7982432 | G | A | -0.48 | 0.038 | 9.8E-36 |
| rs1295685 | 5 | 131996445 | A | G | -0.23 | 0.027 | 2.1E-17 |
| rs12630906 | 3 | 112400097 | C | T | 0.20 | 0.026 | 3.9E-14 |
| rs6780220 | 3 | 33087200 | A | C | 0.17 | 0.025 | 2.9E-11 |
| rs438694 | 10 | 64484681 | G | C | 0.20 | 0.031 | 1.2E-10 |
| rs6010620 | 20 | 62309839 | A | G | 0.17 | 0.028 | 7.3E-10 |
| rs6723629 | 2 | 71067853 | A | C | -0.16 | 0.028 | 1.0E-8 |
| rs79243012 | 4 | 87860512 | T | C | 0.21 | 0.037 | 1.3E-8 |
| rs62192898 | 2 | 234114243 | G | C | 0.14 | 0.025 | 2.3E-8 |
| rs2259735 | 20 | 52788314 | T | C | -0.16 | 0.029 | 4.2E-8 |

SNP, single nucleotide polymorphism; SE,standard error.
